# Arterivirus Nsp1 Modulates the Accumulation of Minus-Strand Templates to Control the Relative Abundance of Viral mRNAs

**DOI:** 10.1371/journal.ppat.1000772

**Published:** 2010-02-19

**Authors:** Danny D. Nedialkova, Alexander E. Gorbalenya, Eric J. Snijder

**Affiliations:** Molecular Virology Laboratory, Department of Medical Microbiology, Center of Infectious Diseases, Leiden University Medical Center, Leiden, The Netherlands; Mount Sinai School of Medicine, United States of America

## Abstract

The gene expression of plus-strand RNA viruses with a polycistronic genome depends on translation and replication of the genomic mRNA, as well as synthesis of subgenomic (sg) mRNAs. Arteriviruses and coronaviruses, distantly related members of the nidovirus order, employ a unique mechanism of discontinuous minus-strand RNA synthesis to generate subgenome-length templates for the synthesis of a nested set of sg mRNAs. Non-structural protein 1 (nsp1) of the arterivirus equine arteritis virus (EAV), a multifunctional regulator of viral RNA synthesis and virion biogenesis, was previously implicated in controlling the balance between genome replication and sg mRNA synthesis. Here, we employed reverse and forward genetics to gain insight into the multiple regulatory roles of nsp1. Our analysis revealed that the relative abundance of viral mRNAs is tightly controlled by an intricate network of interactions involving all nsp1 subdomains. Distinct nsp1 mutations affected the quantitative balance among viral mRNA species, and our data implicate nsp1 in controlling the accumulation of full-length and subgenome-length minus-strand templates for viral mRNA synthesis. The moderate differential changes in viral mRNA abundance of nsp1 mutants resulted in similarly altered viral protein levels, but progeny virus yields were greatly reduced. Pseudorevertant analysis provided compelling genetic evidence that balanced EAV mRNA accumulation is critical for efficient virus production. This first report on protein-mediated, mRNA-specific control of nidovirus RNA synthesis reveals the existence of an integral control mechanism to fine-tune replication, sg mRNA synthesis, and virus production, and establishes a major role for nsp1 in coordinating the arterivirus replicative cycle.

## Author Summary

Plus-strand RNA viruses, a major group of plant and animal pathogens, employ a variety of gene expression strategies. In some groups, the genome is translated into a single polyprotein precursor comprising all viral proteins, while the expression of genomes containing multiple open reading frames commonly depends on the production of additional, subgenomic mRNAs. These serve to translate the open reading frames that are inaccessible to host cell ribosomes engaged in genome translation. Arteriviruses and coronaviruses secure the expression of their structural protein genes by generating an extensive nested set of subgenomic mRNAs, which are copied from a set of complementary minus-strand templates. The production of these subgenome-length minus strands involves a unique mechanism of discontinuous RNA synthesis that essentially competes with the production of the full-length minus strand, the template for genome replication. We describe here that arterivirus non-structural protein 1 (nsp1) modulates the accumulation of minus-strand RNAs to control the relative abundance of both genome-length and subgenomic mRNAs, thereby ensuring efficient production of new virus particles. We found that specific nsp1 mutants with imbalanced mRNA levels and low virus production rapidly acquire additional nsp1 mutations that rescue these defects. Thus, a single arterivirus protein plays a decisive role in the integral control of replication, sg mRNA synthesis, and virus production.

## Introduction

Plus-strand RNA (+RNA) viruses are ubiquitous pathogens of plants, animals, and humans. The translation of their messenger-sense RNA genome yields the core viral enzymes that always include an RNA-dependent RNA polymerase (RdRp) and assemble into a cytoplasmic machinery for viral RNA synthesis. Many +RNA virus groups employ polycistronic genomes and different mechanisms to express genes located downstream of the 5′-proximal open reading frame (ORF). One of these mechanisms involves the synthesis of subgenomic (sg) mRNAs (referred to as “transcription” in this paper). Although the sg mRNAs of +RNA viruses are invariably 3′-coterminal with the viral genome, diverse +RNA viruses have evolved different mechanisms for their production [1].

**Figure 1:**
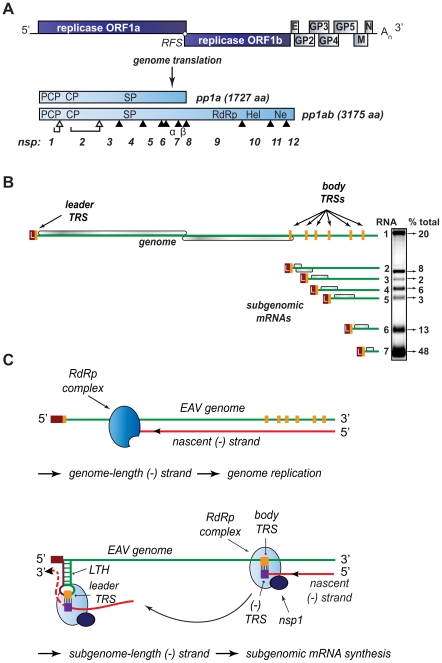
Organization and expression of the polycistronic EAV +RNA genome. (A) Top: EAV genome organization, showing the 5′-proximal replicase open reading frames (ORFs), as well as the downstream ORFs encoding the viral structural proteins envelope (E), membrane (M), nucleocapsid (N), and glycoproteins (GP) 2–5 and the 3′ poly(A) tail (A_n_). Bottom: overview of the pp1a and pp1ab replicase polyproteins that result from genome translation, which requires an ORF1a/1b ribosomal frameshift (RFS) to produce pp1ab. Arrowheads represent sites cleaved by the three virus-encoded proteases (open for autoproteolytically processed ones, closed for sites processed by the main proteinase in nsp4). The resulting nonstructural proteins (nsp) are numbered. The key viral enzymatic domains such as the nsp1 papain-like cysteine proteinase β (PCP), nsp2 cysteine proteinase (CP), nsp4 serine proteinase (SP), nsp9 viral RNA-dependent RNA polymerase (RdRp), nsp10 helicase (Hel), and nsp11 endoribonuclease (Ne) are indicated. (B) Overview of viral mRNA species produced in EAV-infected cells. The ORFs expressed from the respective mRNAs are shown in gray, and the 5′ leader sequence is depicted in dark red. The orange boxes indicate the positions of transcription-regulating sequences (TRS). The gel hybridization image on the right is representative of the wild-type accumulation levels of the seven EAV mRNAs at the time point used for analysis in the study (see text for details). The amount of each mRNA, determined by quantitative phosphorimager analysis, is indicated as percentage of the total amount of viral mRNA. (C) Model for EAV replication and transcription. Continuous minus-strand RNA synthesis yields a genome-length minus strand template for genome replication, a process for which nsp1 is dispensable. Discontinuous minus-strand RNA synthesis results in a nested set of subgenome-length minus strands that serve as templates for sg mRNA synthesis (see text for details). Nsp1 is crucial for this process, which is also guided by a base pairing interaction between the TRS complement [(−)TRS] at the 3′ end of the nascent minus-strand and the genomic leader TRS, present in a RNA hairpin structure (LTH).

The order *Nidovirales* comprises several clades of distantly related enveloped +RNA viruses, including the arteri- and coronavirus families, which infect a wide variety of hosts, ranging from invertebrates to humans. Human coronaviruses are associated with respiratory disease (including severe acute respiratory syndrome (SARS), reviewed in [2]) and arteriviruses like porcine reproductive and respiratory syndrome virus (PRRSV) are important veterinary pathogens. Members of the nidovirus order are characterized by their exceptional genetic complexity, and the group includes the virus families with the largest RNA genomes described to date (25–32 kb). Nidoviruses share important traits in their genome organization and gene expression mechanisms, and their key replicative enzymes are presumed to be evolutionarily related (for a review, see [3]). Their polycistronic genomes are 5′-capped, 3′-polyadenylated, and the two 5′-most open reading frames (ORFs) – ORF1a and ORF1b, encode the viral replicase subunits segregated in two large replicase polyproteins, pp1a and pp1ab, the expression of the latter controlled by a −1 ribosomal frameshift (Fig. 1A). Autoproteolytic processing of these precursors generates between 13 and 16 non-structural proteins (nsps) that direct viral RNA synthesis. Besides genome replication, arteri- and coronavirus RdRp-containing complexes also mediate the synthesis of a distinctive nested set of sg mRNAs that are both 5′- and 3′-coterminal with the viral genome and hence consist of sequences that are noncontiguous in the genomic RNA (Fig. 1B).

Despite recent advances in the structural and functional characterization of individual replicase subunits, the molecular details of nidovirus replication and gene expression remain poorly understood. Studies with nidovirus model systems such as equine arteritis virus (EAV), the arterivirus prototype, have provided some critical insights about viral replicase functions and the regulation of RNA synthesis in infected cells. EAV replicase pp1a and pp1ab are co- and post-translationally cleaved into 13 nsps by viral proteases residing in nsp1, nsp2, and nsp4. The seven viral structural proteins, which are all dispensable for replication and transcription [4], are encoded in a set of overlapping ORFs located in the 3′-proximal quarter of the genome (Fig. 1A). In the six sg mRNAs used to express these ORFs, a common “leader” sequence representing the 5′-proximal 206 nucleotides of the genome is linked to different “body” segments that are co-linear with the 3′-proximal part of the genome (Fig. 1B).

According to the widely supported model proposed by Sawicki and Sawicki [5] (Fig. 1C), the structure of the arterivirus and coronavirus sg mRNAs derives from a discontinuous step during minus-strand RNA synthesis, which is guided by specific RNA signals and resembles copy-choice RNA recombination [6]–[8]. Conserved transcription-regulating sequences (TRS; core sequence 5′ UCAACU 3′ in EAV) precede each structural protein ORF (body TRSs). The same sequence motif is also present at the 3′-end of the genomic leader sequence (leader TRS). Minus-strand RNA synthesis, initiated at the 3′-end of the viral genome, is presumably attenuated at one of the body TRS regions (Fig. 1C; reviewed in [9],[10]). Subsequently, the nascent minus strand, carrying the body TRS complement at its 3′end, is translocated to the 5′-proximal region of the genomic template. During this step, the genomic leader TRS serves as a base-pairing target for the 3′ end of the nascent minus strand, a role that is facilitated, in the case of EAV, by its presence in the loop of an RNA hairpin [11]. When minus strand synthesis resumes, nascent strands are extended with the complement of the genomic leader sequence, yielding a nested set of subgenome-length minus-strand templates that are used for the subsequent synthesis of the various sg mRNAs. If attenuation does not occur, minus-strand RNA synthesis proceeds to yield a full-length complement of the genome, the intermediate required for its replication.

**Figure 2:**
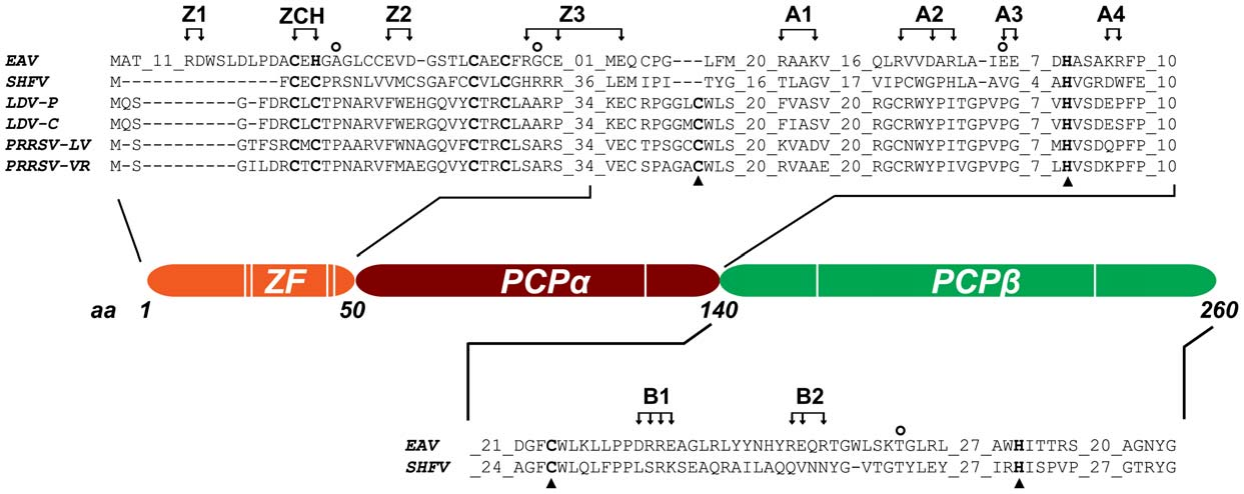
Domain organization of EAV nsp1. The partial sequence alignment shows key regions in the three subdomains previously identified in the arterivirus nsp1 region. GenBank accession numbers for the full-length arterivirus genomes used for the alignment are as follows: EAV, NC_002532; simian hemorrhagic fever virus (SHFV), NC_003092; lactate dehydrogenase-elevating virus (LDV-P and LDV-C), NC_001639 and NC_002534; PRRSV-LV, M96262.2; PRRSV-VR, AY150564. Zinc-coordinating residues are indicated in bold font; the active-site Cys and His of PCPα and PCPβ are indicated with triangles (note the loss of the active-site Cys in EAV PCPα). The positions of amino acid clusters mutated in this study are indicated with arrows. All substitutions were with Ala, with the exception of the ZCH construct, in which Cys-25 and His-27 were swapped. The positions of mutations found in pseudorevertants are indicated with open circles.

Clearly, the protein and RNA factors that determine whether a nidovirus RdRp complex operates in continuous or discontinuous mode, i.e. produces a full-length or a subgenome-length minus strand, must be critical for the coordination of the nidovirus replicative cycle. As in other nidoviruses, the EAV genomic RNA (RNA1) and sg mRNAs (RNA2–RNA7) accumulate in specific molar ratios (see Fig. 1B) that are essentially constant until the peak of viral RNA synthesis is reached [12]. The relative abundance of the transcripts is presumably dictated by the “attenuation rate” at each of the successive body TRSs encountered during minus-strand synthesis, which is primarily determined by the base-pairing potential between the leader TRS and the body TRS complement in the nascent minus strand. Also the sequence context of body TRS motifs and their proximity to the genomic 3′end, which is reflected in the “gradient” of sg RNA sizes, can influence the accumulation of viral RNA species. The importance of TRS-driven RNA-RNA interactions and the potential for a regulatory role of higher order RNA structures was outlined above (reviewed in [9],[10]). At the protein level, however, only a single nidovirus protein specifically involved in transcription was identified thus far: EAV nsp1 was found to be essential for sg mRNA production, while being dispensable for genome replication [13],[14]. Remarkably, nsp1 is also the first protein expressed during infection: it is co-translationally released from the nascent replicase polyproteins by a papain-like cysteine proteinase activity (PCPβ) in its C-terminal domain (Fig. 2). Comparative sequence analysis identified two additional conserved domains: a second, proteolytically silent PCP domain that is functional in other arteriviruses (PCPα; [15]), and an N-terminal zinc finger (ZF) domain [13],[16] that is critical for transcription and efficient production of infectious progeny [13],[14]. Since the accumulation of all sg mRNAs was blocked in the absence of nsp1, the protein was proposed to control a switch between replication and transcription [13].

We have now explored the key regulatory roles of nsp1 in the EAV replicative cycle in unprecedented detail. Our results indicate that in addition to the ZF region, both PCP subdomains of nsp1 are essential for transcription, and suggest an additional role of PCPα in virus production. We also established that nsp1 modulates viral RNA accumulation in an mRNA-specific manner, and thus maintains the balance among the seven viral mRNAs, including the genome. Our data suggests that nsp1 does so by controlling the levels of the full-length and subgenome-length minus-strand templates required for viral mRNA synthesis. The results we obtained from detailed characterization of nsp1 mutants and pseudorevertants provided compelling evidence for a close link between the regulation of individual nidovirus mRNA levels and the efficient production of infectious progeny.

## Results

### Rationale for mutagenesis of EAV nsp1

Previous studies of the role of nsp1 in the EAV replicative cycle focused on the conserved amino acids presumed to be essential either for zinc binding by the ZF domain or for the catalytic activity of the PCPβ autoprotease [14]. Mutations that blocked the release of nsp1 from the replicase polyproteins were lethal, likely due to their interference with downstream polyprotein processing steps that are essential for genome replication [17]–[19]. By contrast, replacements of putative zinc-coordinating residues either selectively abolished transcription of all viral sg mRNAs or interfered with virus production without affecting viral mRNA accumulation. In an attempt to expand our repertoire of viable nsp1 mutants, we now used two approaches: i) alanine scanning mutagenesis of non-conserved clusters of polar residues found throughout the nsp1 sequence, and ii) a Cys↔His interchange at the positions of residues Cys-25 and His-27, which have both been implicated in zinc coordination [13],[16]. The first approach is less likely to perturb the protein's overall stability, since clusters of charged residues are usually found on the protein surface, where they may mediate interactions with other biomolecules via electrostatic interactions or hydrogen bond formation [20]–[22]. We reasoned that the second approach might preserve zinc coordination but could nevertheless have a subtle effect on zinc binding that might be translated in a measurable effect on one or more of nsp1's functions. Moreover, if these substitutions would compromise virus replication, isolation of revertant viruses encoding compensatory second-site mutations might reveal potential regulatory protein-protein or protein-RNA interactions.

Table 1 lists the nsp1 mutants characterized in this study. In all three subdomains of nsp1, clusters of two or three charged amino acids within a five- to seven-amino acid stretch were substituted with Ala. Constructs with Ala replacements in the ZF domain were designated Z (1 to 3), while those with replacements in the PCPα and PCPβ domains were designated A (1 to 4) and B (1 and 2), respectively (see Table 1 and Fig. 2). In addition, we swapped the Cys-25 and His-27 residues to generate the ZCH mutant. Full-length RNA transcribed from EAV cDNA clones encoding these nsp1 mutations was transfected into BHK-21 cells. Analysis of nsp1 mutant phenotypes was performed during the peak of viral RNA synthesis and before the bulk of infectious progeny was produced by the wild-type (wt) control (11 h post-transfection; referred to as first-cycle analysis). Previous studies of intracellular viral RNA levels had been hampered by the considerable variability in transfection efficiencies of synthetic EAV full-length RNAs. For comparison between mutants, these studies used the genomic RNA as an internal standard for each sample to calculate relative ratios of viral mRNA accumulation levels [6],[14]. Using an improved electroporation protocol (for details, see Materials and Methods), we now achieved very consistent and relatively high RNA transfection efficiencies (between 45% and 55% of positive cells at 11 h post-transfection with replication-competent synthetic EAV RNAs; data not shown). This allowed for the comparison of the absolute levels of mRNA accumulation and the detailed first-cycle analysis of EAV nsp1 mutants at a time point at which differences in virus production or (pseudo)reversion would not influence the assessment of their phenotype.

**Table 1:** Overview of the genotype and first-cycle phenotype of EAV nsp1 mutants described in this study.

Construct	Genotype	Mutant codons^a^	Replication^b^	Transcription^b^
pEAV211	wt	NA^c^	+++	+++
Z1	R15A D16A	267- ***GC***G G***C***C-272	+++++	−
ZCH	C25H H27C	297- ***CA***U ***UG***U-305	+++	++++
Z2	E34A D36A	324-G***C***A G***C***C-332	++++	−
Z3	R46A E49A E52A	360- ***GC***C G***C***A G***C***G-380	+++++	−
A1	R80A K83A	462- ***GC***A ***GC***A -474	+++++	++
A2	R103A D106A R108A	531- ***GC***U G***CA*** ** ***GC***G-548	+++++	−
A3	E112A E113A	558-G***CC*** G***C***G-563	+++	+++
A4	K126A R127A	600- ***GCA*** ** ***GC***U-605	++++	+++
B1	D172A R173A R174A E175A	738-G***CU*** ** ***GCA*** ** ***GC***U G***C***G -749	−	−
B2	R186A E187 R189A	780- ***GC***C G***C***A ***GC***G-791	+++++	−

a Nucleotide substitutions are indicated in bold italics; numbers indicate the start and end coordinates of the mutated codons in the EAV genome.

b Transfected cells were analyzed by IFA and gel hybridization analysis at 11 h post-transfection. Wild-type levels of replication and transcription (based on accumulation levels of genomic and sg mRNAs, respectively) are indicated with +++. A 2- to 4-fold increase in genomic RNA levels as compared to wild-type is denoted by ++++, while an increase of >4-fold is shown as +++++. Likewise, a >2-fold increase or decrease in accumulation levels of at least two sg mRNA species is shown as ++++ and ++, respectively (see text for details).

c NA, not applicable.

### All nsp1 subdomains are critical for transcription, while the ZF and PCPα domains are also important for virus production

**Figure 3:**
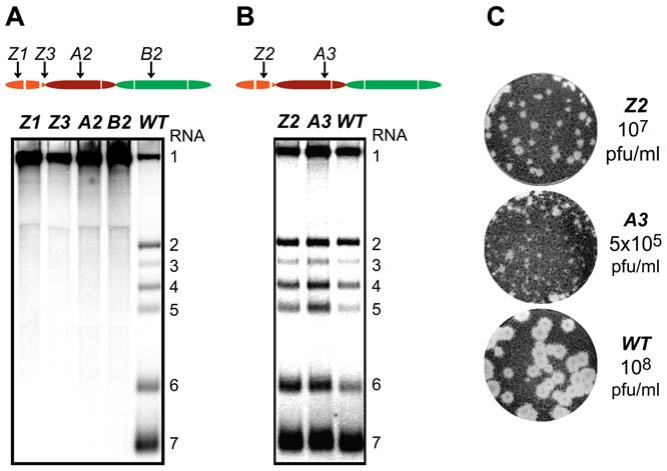
Importance of nsp1 subdomains for transcription and virus production. (A, B). Analysis of EAV-specific mRNA accumulation by gel hybridization. The domain organization of nsp1 is depicted as in Fig. 2 and the positions of the clusters of amino acid mutations analyzed are indicated with arrows. BHK-21 cells were transfected with RNA transcribed from wt or selected mutant EAV full-length cDNA clones. Total intracellular RNA was isolated at 11 h post-transfection and resolved by denaturing formaldehyde electrophoresis. Equal loading of samples was confirmed by ethidium bromide staining of ribosomal RNA (data not shown). EAV-specific mRNAs were detected by hybridization of the gel with a ^32^P-labelled probe complementary to the 3′-end of the viral genome and subsequent phosphorimaging. The positions of the EAV genome (RNA1) and the six sg mRNAs (RNA2 to RNA7) are indicated. (C) Plaque phenotype and virus titers of the Z2 and A3 mutants. Plaque assays were performed on BHK-21 using cell culture supernatants harvested 24 h after transfection. Cells were incubated under a semi-solid overlay at 39.5°C for 72 h, fixed and stained with crystal violet. Virus titers represent an average of three independent experiments. Pfu, plaque-forming units.

The ZF domain of nsp1 is essential for sg mRNA production [13],[14], but the question of whether the PCPα and PCPβ domains also contribute to the protein's function in transcription has not been previously addressed, partly due to the nonviable phenotype of PCPβ mutants in which the nsp1/2 cleavage was impaired [14]. Consequently, we first analyzed the impact of clustered charged-to-alanine replacements in nsp1 on viral mRNA accumulation. Cells transfected with nsp1 mutants were harvested 11 h after transfection, intracellular RNA was isolated and resolved in denaturing gels, and viral mRNAs were detected by hybridization to a probe complementary to the 3′-end of the genome and thus recognizing all viral mRNAs. Substitutions in the ZF domain (mutant Z1), as well as in the region connecting the ZF and PCPα domains (Z3), the PCPα domain itself (A2) and, notably, also the PCPβ domain (B2) rendered viral sg mRNAs undetectable. In addition, all four mutants displayed a noticeable increase in genomic RNA levels (Fig. 3A). By contrast, accumulation of all viral mRNAs was blocked in the B1 mutant (Table 1 and data not shown), possibly due to the proximity of the charged cluster to the active site Cys of PCPβ (Fig. 2). These results demonstrate that all subdomains of nsp1, including PCPβ, are important for transcriptional control. This novel role of the PCPβ domain seems to be genetically separable from its autoproteolytic activity.

We previously reported that certain substitutions of proposed nsp1 zinc-coordinating residues considerably reduced the yield of infectious progeny without noticeably affecting viral RNA accumulation [14]. This phenotype was also observed in this study for mutants Z2 (ZF domain) and A3 (PCPα domain), in which clusters of alanine substitutions were introduced. These had no apparent effect on viral mRNA levels (Fig. 3B), while progeny virus titers were reduced by 10- and 200-fold, respectively (Fig. 3C), in supernatants harvested 24 h after transfection, well beyond the time point of maximum virus production by the wt control (data not shown). Accordingly, plaques of the Z2 mutant were somewhat smaller than those of the wt virus, and those of the A3 mutant were minute (Fig. 3C). Titers and plaque phenotypes remained essentially unchanged at 48 h post-transfection, arguing against a delay in virus production. Sequence analysis of the A3 progeny revealed reversion of the E113A mutation to the wt sequence at later time points (data not shown). These observations imply that both the ZF and the PCPα domains of EAV nsp1 are involved in a step of the viral replicative cycle that is downstream of transcription and is critical for the efficient production of infectious virus particles.

### Mutations in nsp1 can differentially affect accumulation of viral mRNA species

**Figure 4:**
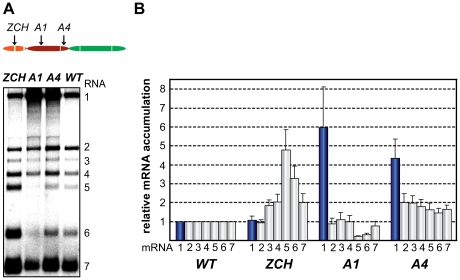
Multiple mutations in nsp1 exert species-specific effects on viral mRNA accumulation. (A, B) Gel hybridization analysis and quantification of EAV-specific mRNA accumulation in cells transfected with the ZCH, A1, A4 mutant or a wt control. (A) Viral mRNA accumulation was analyzed at 11 h post-transfection by gel hybridization as described in the legend to Fig. 3. (B) The accumulation levels of each viral mRNA in the nsp1 mutants were quantified by phosphorimaging in the linear range of exposure and normalized to the level of accumulation of each corresponding viral mRNA in the wt control, which was set at 1. Genomic RNA levels are represented as blue bars. The relative values correspond to the means from three independent transfections and error bars denote standard deviation.

Two mutant phenotypes were previously described upon examination of the role of EAV nsp1 in transcription: one in which sg mRNA accumulation was selectively abolished, and another in which the levels of all sg mRNAs were uniformly reduced relative to that of the genomic RNA [13],[14]. In this study, the ZCH, A1, and A4 mutants displayed a third phenotype, demonstrating that replacements in nsp1 can affect EAV RNA levels in an mRNA-specific manner. The swapping of two proposed zinc-coordinating residues in the ZCH mutant resulted in the upregulation of a subset of sg mRNAs. In comparison to the wt control, the accumulation levels of RNA3, 4, 5, 6 and 7 were increased, while those of the viral genome and RNA2 remained largely unchanged (Fig. 4, A and B). The increase in mRNA levels was not uniform, being more pronounced for RNA5 and RNA6 (4.5-fold and 3-fold, respectively) than for RNAs 3, 4, and 7 (∼2-fold). Also, the substitution of two positively charged residues in the PCPα domain of the A1 mutant (see Fig. 2) resulted in reduced accumulation levels of RNA5 and RNA6 (3- to 4-fold), and RNA7 (∼30%), but not of RNAs 2 to 4 (Fig. 4, A and B). In contrast, genomic RNA accumulation was dramatically enhanced in the A1 mutant (Fig. 4, A and B). This aspect of the mutant phenotype had been previously described in mutants that did not produce any sg mRNAs [14], in which it was attributed to the increased availability of key factors for viral replication. This explanation does not seem likely for the A1 mutant, however, in which accumulation of only two of the six viral sg mRNAs was reduced (Fig. 4). Furthermore, another PCPα mutant - A4 (see Fig. 2), displayed 4-fold higher levels of genome accumulation without any significant decrease in sg mRNA production (Fig. 4, A and B).

Since EAV mRNAs accumulate to different levels in molar ratios that are maintained through most of the replicative cycle (see Fig. 1B), we will refer to these levels in the wt control as “balanced”. By contrast, the ZCH, A1, and A4 mutants exerted differential effects on viral mRNA abundance, resulting in “imbalanced” viral mRNA accumulation profiles. The unusual phenotypes of these mutants implicated nsp1 in the mRNA-specific modulation of viral RNA levels, and prompted us to investigate their molecular basis in greater detail.

### Accumulation of viral mRNAs and their corresponding minus-strand RNA templates are similarly affected by nsp1 mutations

According to the current consensus in the field, minus-strand RNA synthesis in arteri- and coronaviruses can operate in either continuous or discontinuous mode, generating genome- or subgenome-length templates respectively [5],[9],[10]. The relative abundance of the corresponding minus-strand template presumably determines the level to which each of the viral mRNAs accumulates [23],[24].We therefore sought to determine whether the differential effects of nsp1 mutations on the accumulation of EAV mRNA species were accompanied by changes in the levels of the corresponding minus-strand templates. To this end, we developed an RNase protection assay for the detection and quantification of EAV minus-strand RNA species. We employed a two-step protocol in which total RNA extracted from cells transfected with nsp1 mutants was first denatured and self-annealed. Due to the large excess of plus strands present in RNA samples extracted from EAV-infected cells [25], all minus strands are expected to be present in duplexes after this annealing step, facilitating their subsequent reliable quantification. The remaining single-stranded RNA was then removed by RNase T1 digestion. Following inactivation of the enzyme, we added an excess of ^32^P-labeled transcripts of positive polarity, which were derived either from a region unique to the EAV genome, or from the leader-body junction regions of RNA6 and RNA7. The samples were then subjected to a second round of RNA denaturation, hybridization, and digestion with RNase A and T1, after which the protected fragments were analyzed by electrophoresis.

**Figure 5:**
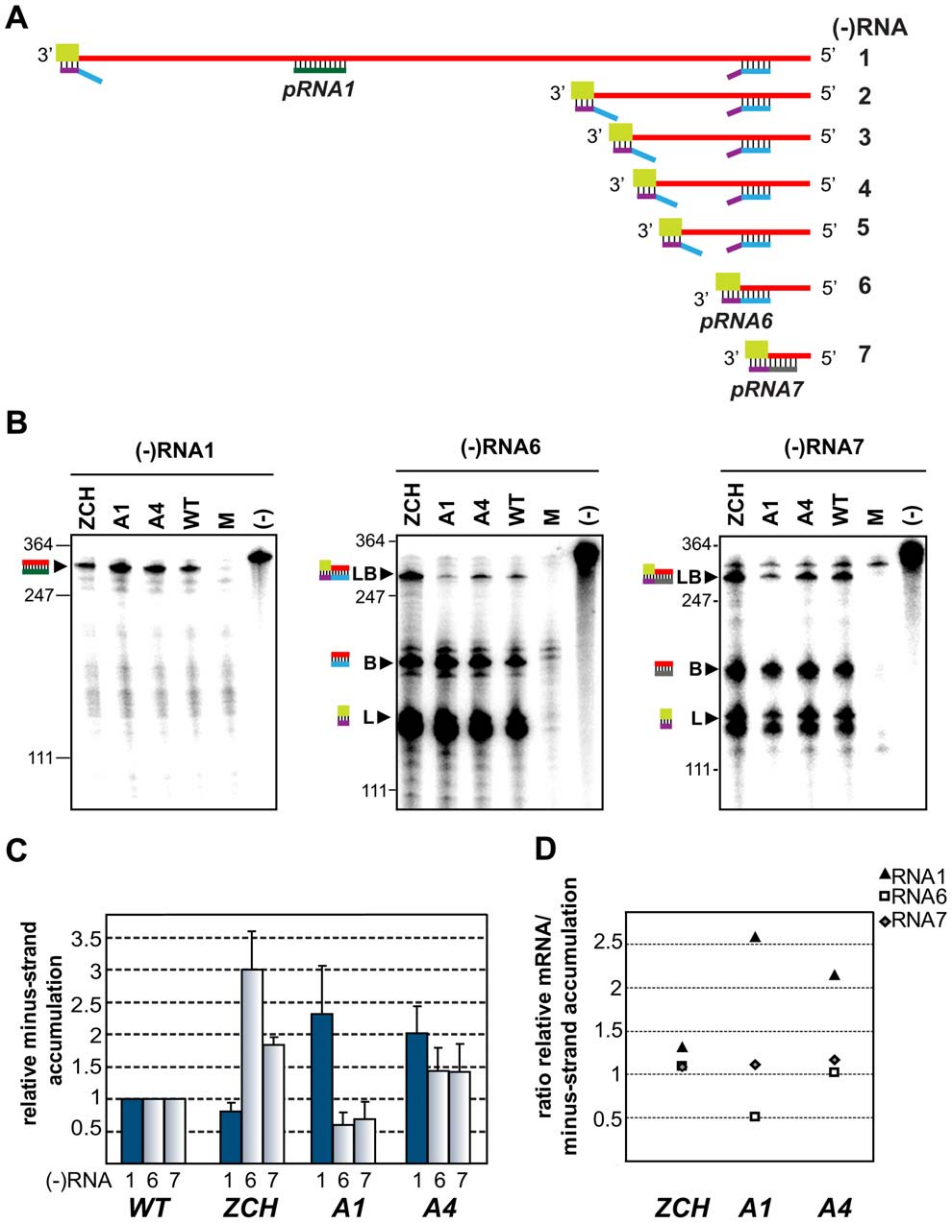
Minus-strand RNA accumulation is also modulated by mutations in nsp1. (A–D) Analysis and quantification of EAV minus-strand accumulation by a two-cycle RNase protection assay. (A) Schematic representation of the nested set of viral minus-strand RNA [(−)RNA] species produced in EAV-infected cells. The anti-leader sequence is depicted in light green. The in vitro-transcribed plus-strand probes used for detection of (−)RNA1 (pRNA1), (−)RNA6 (pRNA6) and (−) RNA7 (pRNA7) are shown. pRNA6 and pRNA7 target the leader-body junction sequences of (−)RNA6 and (−)RNA7, respectively. Note that hybridization with pRNA1 results in the protection of a single fragment, while the probes for (−)RNAs 6 and 7 each protect three fragments – one derived from the full-length sg minus strand, and two fragments derived in part from partial hybridization of these probes to larger viral (−)RNAs in which the target sequences are noncontiguous (exemplified for pRNA6). For simplicity, non-EAV sequences present near the termini of the three probes were omitted from the scheme. (B) Viral (−)RNA accumulation was analyzed at 11 h post-transfection for the ZCH, A1 and A4 mutants, and a wt control. Protected fragments were resolved on denaturing 5% polyacrylamide/8M urea gels and visualized by phosphorimaging. The constructs analyzed are labeled above the lanes (M, mock-transfected cells; (−), no-RNase control that shows a band corresponding to 0.2 fmol of the full-length probe). Sizes (nt) of RNA markers have been indicated on the left. The single 327-nt protected fragment resulting from hybridization with the positive-sense probe for RNA1(−) is indicated. The probes for subgenome-length minus strands protected fragments derived from the full-length (−)RNA6 and (−)RNA7 (327 nt and 319 nt, respectively; denoted with LB), as well as from the (−)RNA6 and (−)RNA7 body sequences (188 nt and 180 nt, respectively; denoted with B) and the anti-leader sequence (139 nt; denoted with L). The presence of two bands in the size range of the anti-leader fragment has been described previously [55]. (C) The relative levels of minus-strand accumulation were quantified by phosphorimaging. For (−)RNAs 6 and 7, only the bands resulting from protection of full-length sg minus strands (denoted with LB in panel [B]) were quantified. The values correspond to the means from three independent transfections that were normalized to the level of accumulation of each minus-strand RNA in the wt control, which was set at 1. Intracellular RNA from the same transfection samples for which plus-strand accumulation was quantified (Fig. 4B) was used. Genomic minus-strand RNA levels are represented as dark blue bars. Error bars denote standard deviation. (D) The ratio of plus-strand to minus-strand accumulation for RNAs 1, 6 and 7 was calculated using the mean relative values obtained in Fig. 4B and Fig. 5C.

The minus-strand templates of the most abundant viral mRNAs – RNA1, 6, and 7, were selected for quantitative analysis. Accumulation levels of genome-length [(−)RNA1] and subgenome-length minus strands corresponding to RNA6 and RNA7 [(−)RNA6 and (−)RNA7] were quantified in total intracellular RNA extracted at 11 h post-transfection with the ZCH, A1, and A4 mutants, and a wt control. Subgenomic minus- and plus-strand levels were similarly affected by the nsp1 mutations in a sg RNA-specific manner (Fig. 5). Genomic minus-strand accumulation was increased in the A1 and A4 mutants, albeit to a somewhat lesser extent as compared to the increase in genomic plus-strands (Fig. 4). These results clearly implicate nsp1 in a regulatory step (or steps) that controls minus-strand RNA accumulation, and ultimately determines the levels to which both genome- and subgenome-length mRNA species accumulate in EAV-infected cells.

### Changes in the accumulation of viral mRNAs induced by nsp1 mutations result in altered viral protein levels

**Figure 6:**
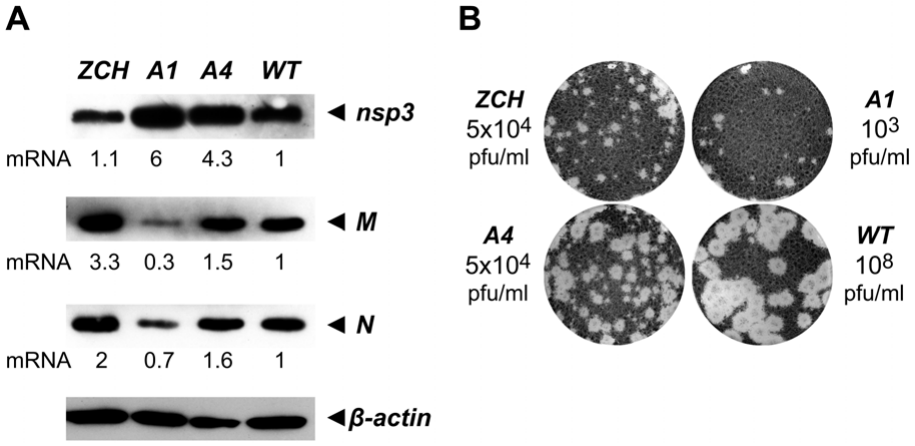
Analysis of viral protein accumulation and virus production by selected nsp1 mutants. (A) Western blot analysis of EAV-specific protein accumulation. Cells transfected with wt or mutant viral genomes were harvested 11 h after transfection and equal amounts of total protein were analyzed with EAV-specific sera detecting nsp3, M and N, which are translated from RNAs 1, 6 and 7, respectively. The relative levels of each mRNA template (derived from Fig. 4B) are indicated below the gels. Beta-actin was used as a loading control. (B) Plaque phenotype and virus titers of the ZCH, A1 and A4 mutants in comparison with wt. Plaque assays were performed on BHK-21 using cell culture supernatants harvested 24 h after transfection. Virus titers represent an average of three independent experiments.

The relative abundance of nidovirus mRNAs likely serves to regulate the relative concentration of their respective translation products during infection. It was therefore important to determine whether viral protein levels indeed mirrored the specific changes in viral mRNA levels caused by mutations in nsp1. To this end, we examined the intracellular accumulation of the EAV replicase subunit nsp3, and the structural proteins M and N, in cell lysates harvested 11 h after transfection (Fig. 6A). When compared to the wt control, nsp3 was more abundant in cells transfected with the A1 and A4 mutants, in line with the increased genome levels observed for these mutants at the same time point (Fig. 4). The intracellular levels of the M and N proteins were also in general agreement with the abundance of their corresponding mRNA templates (RNA6 and RNA7, respectively). Interestingly, even the modest reduction in RNA7 levels detected for the A1 mutant (∼30%, Fig. 4B) was reflected in a decrease in N protein levels (Fig. 6A). The close correlation between mRNA and corresponding protein levels argues against the possibility that the engineered nsp1 mutations might have caused a defect in viral mRNA translation. Overall, these data establish that even modest changes in viral mRNA accumulation are directly translated into altered viral protein levels during EAV infection.

### Production of infectious virus particles is severely impaired in nsp1 mutants with imbalanced mRNA accumulation

We next examined whether the production of infectious virus particles was disturbed in the three nsp1 mutants with imbalanced mRNA accumulation profiles. EAV assembly involves the coordinated interplay of the N protein and six envelope proteins, all of which are required for virus infectivity [4],[26]. A critical step in assembly is the heterodimerization of the major viral envelope proteins, GP_5_ and M [27],[28], and both the GP_5_ and M protein levels, as well as the levels of their mRNA templates, RNA5 and RNA6, were strongly affected in the ZCH and A1 mutants (Fig. 4B and 6A). Furthermore, other documented interactions, such as the oligomerization of the minor envelope proteins - GP_2b_, GP_3_ and GP_4_ [29], could be readily affected by the altered relative abundances of viral structural proteins. The decreased ratio of N protein and genomic RNA in the A1 and A4 mutants (Fig. 4B and Fig. 6A) could also adversely affect the assembly of infectious virions. Analysis of the infectious progeny virus titers in culture supernatants harvested 24 h after transfection with the ZCH, A1, and A4 mutants indeed revealed a dramatic loss of infectivity (Fig. 6B). In comparison to wt, the infectious progeny yield of the ZCH and A4 mutants were reduced by approximately 4 logs and that of the A1 mutant by ∼5 logs.

We subsequently purified virions by sedimentation through a sucrose cushion in order to quantify their genomic RNA content by reverse transcription-quantitative PCR. Virions from medium harvested at 24 h after transfection with the ZCH, A1, and A4 mutants were compared with the progeny produced by the wt control. Consistent with the reduction in infectious progeny titers, these results showed a decrease in the total number of genome-containing virus particles secreted from cells transfected with each of the three mutants (Table 2). Interestingly, when the relative specific infectivity of each mutant virion preparation was assessed by relating the genomic RNA content to the plaque-forming units (pfu), a marked decrease in the pfu per unit of genomic RNA ratio of the ZCH, A1, and A4 virion preparations was revealed (Table 2). The ZCH, A1, and A4 mutations thus seem to affect both the total number of secreted virions, as well as their specific infectivity. Also, even at this relatively early time point post transfection, the three mutants exhibited heterogeneous plaque morphology, indicative of rapid reversion (Fig. 6B). These results, together with the RNA and protein analyses outlined above (Fig. 4B and 6A), demonstrate that even moderate changes in the accumulation of EAV mRNAs and proteins can be associated with a dramatic decrease in the yield of infectious progeny. Thus, a previously unnoticed link seems to exist between the fine-tuning of the relative abundance of EAV mRNAs (and, consequently, viral protein levels) and the efficiency of virion biogenesis.

**Table 2:** Relative specific infectivities of virus particles from nsp1 mutants with imbalanced mRNA profiles.

Construct	Relative infectivity^a^		Relative genomic RNA content^b^		Relative specific infectivity^c^	
	Exp. 1^d^	Exp. 2	Exp. 1	Exp. 2	Exp. 1	Exp. 2
pEAV211	1	1	1	1	1	1
ZCH	4×10^−4^	5×10^−4^	2.7×10^−1^	7.9×10^−1^	1.9×10^−3^	5.1×10^−4^
A1	2×10^−5^	5×10^−6^	8.5×10^−4^	6.4×10^−3^	2.4×10^−2^	7.9×10^−4^
A4	4×10^−4^	4×10^−4^	1.8×10^−2^	1.6×10^−1^	2.2×10^−2^	2.4×10^−3^

a Virus titers were determined by plaque assays of culture supernatants harvested at 24 h post-transfection and normalized to the pfu/ml value of the wt control, which was set at 1.

b EAV genomic RNA levels were quantified in virion preparations obtained from culture supernatants harvested at 24 h post-transfection by reverse transcription and quantitative PCR. Values for mutant virions were obtained by comparing their threshold cycle (Ct) against the qPCR standard curve, and were normalized relative to the genomic RNA level in the wt control, which was set at 1.

c Relative specific infectivity values were calculated by dividing the relative infectivity (mutant∶wt pfu ratio) by the relative genomic RNA content for each construct.

d The data shown are derived from two independent experiments.

### Second-site mutations in nsp1 can restore both the quantitative balance among viral mRNA species and efficient virus production

To gain more insight into the molecular basis of the nsp1 mutant phenotypes described above, we attempted to isolate revertant viruses encoding compensatory second-site mutations. The mutants in which sg mRNA accumulation was completely blocked, however, proved to be extremely stable. We were repeatedly unable to detect infectious particles in supernatants of transfected cells, even after prolonged incubation (up to 70 h) at 39.5°C or at a reduced temperature of 35°C, or after using these supernatants to infect fresh BHK-21 cells (data not shown). By contrast, the rapid appearance of large plaque variants among the prevailing small plaques produced by the ZCH, A1, and A4 mutants (see Fig. 6B) suggested genetic heterogeneity. Large plaque clones of these three mutants were isolated and propagated in fresh cells, and the EAV nsp1 gene was amplified by RT-PCR. Sequence analysis of the PCR products confirmed the presence of the originally engineered mutant codons, and identified additional mutations in the nsp1-coding sequence in the majority of the plaques analyzed (data not shown). A pseudorevertant of ZCH had acquired a substitution in the vicinity of the original mutations (Ala-29 to Asp). Interestingly, Ala-29 was also mutated in five independent A1 pseudorevertants, where it had been replaced with Lys due to two nucleotide substitutions (Table 3). In addition, four clones of the A4 offspring contained a Thr-196 to Lys substitution, and Gly-47 to Ala and Glu-112 to Lys replacements were found in one clone each.

**Table 3:** Overview of EAV nsp1 pseudorevertants described in this study.

Construct	Second-site mutation^a^	Wild-type codon^b^	Mutant codon	Titer (pfu/ml)^c^	Plaque size
pEAV211	NA^d^	NA	NA	1×10^8^	wild-type
ZCH	A29D (*1*)	309-GCC e	GAC	5×10^6^	intermediate
A1	A29K (*5*)	309-GCG	AAG	5×10^6^	intermediate
A4	G47A (*1*)	363-GGU	GCU	5×10^7^	wild-type
	E112K (*1*)	558-GAA	AAA	5×10^7^	wild-type
	T196K (*4*)	810-ACA	AAA	5×10^7^	wild-type

a Virus clones were isolated from plaque assays of culture supernatants harvested between 20 h and 24 h post transfection. The number of clones containing each mutation is indicated in brackets.

b Numbers indicate the start coordinate of the codon in the EAV genome.

c Pseudorevertants were reconstructed in the wild-type EAV background, and infectious progeny titers were determined by plaque assays of culture supernatants harvested at 24 h post-transfection. The average titers of three independent experiments are shown.

d NA, not applicable.

e A silent mutation changing the original alanine codon from GCG to GCC was introduced as a marker mutation upon construction of the ZCH mutant.

**Figure 7:**
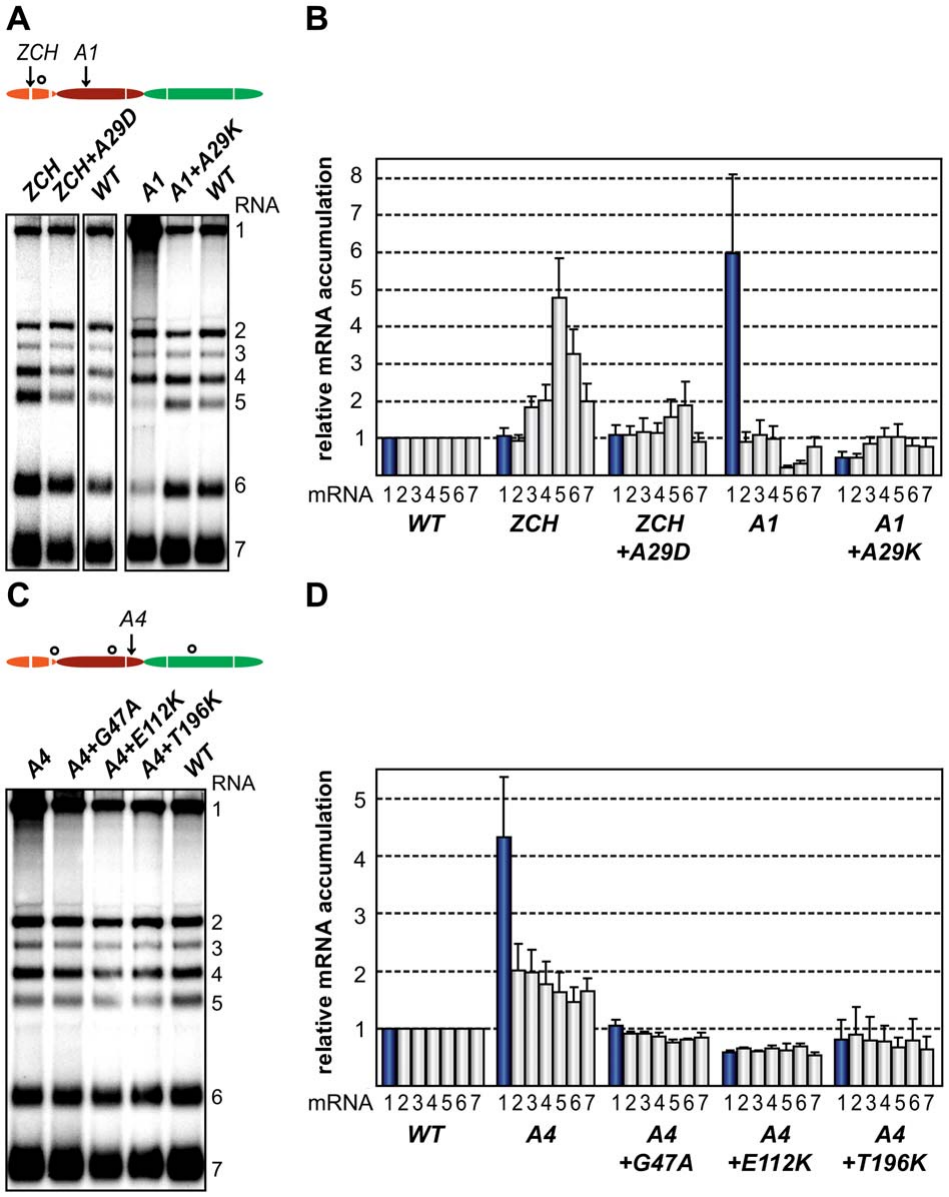
Second-site mutations in nsp1 moderate species-specific defects in mRNA accumulation. (A–D) Gel hybridization analysis and quantification of EAV-specific mRNA accumulation. (A) BHK-21 cells were transfected with ZCH and A1 mutants, reconstructed pseudorevertants, and wt controls. The positions of the originally mutated amino acid clusters are indicated with arrows; the open circle denotes the position of second-site mutations. Viral mRNAs were analyzed 11 h post-transfection by gel hybridization as described above. (B) For ZCH+A29D and A1+A29K, the accumulation levels of each viral mRNA were quantified by phosphorimaging and the values were normalized to the wt level of accumulation of each corresponding viral mRNA from the same experiment, set at 1. Genomic RNA levels are shown as blue bars. The relative values correspond to the means from three independent transfections and error bars denote the standard deviation. The relative accumulation levels of viral mRNAs at 11 h post-transfection for the ZCH and A1 mutants are derived from Fig. 4B and are represented here to facilitate comparison between mutant and pseudorevertant phenotypes. (C) BHK-21 cells were transfected with the A4 mutant, reconstructed pseudorevertants and a wt control. The positions of the originally mutated amino acid cluster and the second-site mutations are indicated as in (A). Viral mRNAs were analyzed 11 h post-transfection. (D) For A4+G47A, A4+E112K, and A4+T196K, quantification of relative viral mRNA accumulation levels was performed as described in (B). Similarly, the relative accumulation levels of viral mRNAs at 11 h post-transfection for the A4 mutant derived from Fig. 4B are represented to facilitate comparison.

To ascertain these second-site substitutions conferred a replicative advantage, they were introduced into their respective parental (mutant) full-length cDNA clones, yielding a set of viruses collectively referred to as “nsp1 pseudorevertants”. Cell culture supernatants harvested 24 h after transfection with RNA from nsp1 pseudorevertants indeed contained between 2 and ∼4 logs more infectious progeny than those of the original mutants (Table 3), confirming the compensatory nature of the second-site mutations. Both virus titer and plaque size of the three viruses carrying a second-site mutation in the A4 mutant background were similar to those of the wt control (Table 3; data not shown). Replacement of Ala-29 with Asp or Lys in the ZCH and A4 backgrounds, respectively, increased virus titers by 2 to ∼4 logs, with plaque sizes being intermediate between those of the parental mutant and the wt control (Table 3; data not shown). The relative specific infectivities of virion preparations derived from all nsp1 pseudorevertants were also considerably higher in comparison to those of the parental mutants (data not shown). Notably, the pseudorevertants showed a partial or complete restoration of the mRNA-specific defects that we had observed for the original mutants (Fig. 7). Introduction of the Ala-29 to Asp substitution in the ZCH mutant was accompanied by a considerable reduction of the otherwise abnormally high accumulation of RNAs 3 to 7, though RNA5 and RNA6 were still present at ∼150% of the normal level (Fig. 7B). Likewise, the Ala-29 to Lys replacement moderated the effect of the A4 substitutions on the accumulation levels of RNA1, RNA5, and RNA6; those of RNA1 and RNA2 were somewhat reduced relative to wt. Introduction of G47A, E112K, or T196K in the A4 background in each case suppressed the increased ratio of genomic to sg mRNA that was characteristic for the parental mutant virus (Fig. 7D). Thus, both the pronounced mRNA-specific accumulation defects, as well as the associated drop in virus production observed for the ZCH, A1, and A4 mutants (Fig. 4) were considerably alleviated by second-site mutations in nsp1. The location of both the original and the second-site mutations in nsp1 revealed multiple genetic interactions between all nsp1 subdomains that are important for the protein's role in regulating the relative abundance of EAV mRNAs. In addition, the increased virus production by the pseudorevertants was invariably correlated with a distinct shift towards an mRNA accumulation profile that was (more) similar to wt (Fig. 7, B and D). These observations provide compelling evidence that efficient virus production depends on maintaining “balanced” viral mRNA levels in EAV-infected cells.

## Discussion

In order to progress through their replicative cycle, +RNA viruses need to orchestrate key macromolecular processes that often overlap in time and possibly also in space. Multifunctional viral proteins, which can regulate and interlink multiple of these steps, may therefore have evolved in order to facilitate this spatio-temporal coordination. EAV nsp1 was first identified as a general transcription factor that is also critical for replicase polyprotein processing and efficient production of infectious progeny [13],[14]. By employing forward and reverse genetics, we have now examined the significance of all nsp1 subdomains for these multiple regulatory roles. Detailed quantitative analyses of viral mRNA species and their corresponding minus-strand templates in nsp1 mutants allowed us to identify a previously unrecognized function of the protein in the mRNA-specific regulation of viral RNA abundance. Nsp1 seems to perform this function by controlling the accumulation of the minus-strand templates for viral mRNA synthesis. Together with its well-documented general role in transcription, these results place nsp1 at the heart of the unique process of discontinuous minus-strand RNA synthesis that is employed by arteriviruses and other nidoviruses. Even the modest changes in viral mRNA levels we observed in nsp1 mutants with differential deviations in viral mRNA abundance are seemingly reflected in altered levels of their translation products. The dramatically reduced virus yields of these mutants revealed that the critical fine-tuning of the “balance” of EAV mRNA accumulation has major implications for the final stages of the replicative cycle. The importance of this balance is greatly emphasized by the rapid emergence of pseudorevertant viruses, in which both the mRNA-specific accumulation defects and the impaired virus production were significantly moderated. The mapping of compensatory second-site mutations to the nsp1 gene itself implies that multiple physical interactions among nsp1 subdomains are essential for the protein's role in transcriptional control. Our findings constitute the first evidence for the involvement of a protein factor in regulating the relative abundance of individual mRNA species during nidovirus infection.

### The intricate interplay between nsp1's subdomains

Previous reports have suggested that the multiple roles of nsp1 during EAV infection can (in part) be functionally separated, with the ZF domain being essential for transcription and efficient virus production, and the PCPβ protease cleaving the nsp1/2 site, irrespective of the ZF integrity [14]. This study extended and refined the above concept showing, first, that the functional repertoire of nsp1 also includes the differential control of mRNA accumulation, and, second, that some functions may be based on the interplay between two or even all three of the protein's subdomains. The phenotypes observed upon replacing charged amino acid clusters with alanine established the importance of all nsp1 subdomains for sg mRNA accumulation (Fig. 3A). The involvement of PCPβ in transcription is seemingly unrelated to its autoproteolytic function, whose inactivation is detrimental to genome replication [14]. The co-translational, *cis*-cleavage of the nsp1/2 site in the nascent replicase polyproteins [30] is probably the sole processing step mediated by PCPβ, which becomes rapidly available to exercise any *trans*-acting, non-proteolytic activities it may have. Such secondary non-proteolytic functions have previously been reported for several +RNA viral autoproteinase domains, such as those of hepatitis C virus NS2 [31] and beet yellow virus L-Pro [32].

The previously uncharacterized PCPα domain, a PCPβ paralog that has lost its proteolytic capacity in EAV [15], appears to cooperate with the ZF in transcription and virion biogenesis (Fig. 3). Likewise, it works in concert with both flanking nsp1 subdomains in controlling the relative abundance of viral mRNAs (Fig. 4, Table 3, and Fig. 7). The Lys and Arg residues replaced with alanine in the A1 and A4 mutants, and the Lys that had evolved in their pseudorevertants are all basic residues. They are found in different nsp1 subdomains, but could well be spatially juxtaposed to provide a positively charged side chain to a functional region that is involved in interactions essential for one or more of the protein's activities. The Gly-47 to Ala reversion, which maps to the junction region between the ZF and PCPα, might serve to reposition these subdomains relative to each other. A similar readjustment of the protein's tertiary structure might account for the compensatory effect exerted by the Ala-29 to Asp reversion on the ZCH mutant. Despite the fact that its orthologs in other arteriviruses have retained their proteolytic activity, it is tempting to speculate that the incorporation of the PCPá domain in the arterivirus nsp1 region can be primarily attributed to the non-proteolytic functions outlined above. On the whole, there seems to be considerable cooperation between nsp1 subdomains. This interplay appears crucial for coupling the different processes that nsp1 controls and may be based on interactions that are either intra- or intermolecular, in view of the protein's ability to form homo-oligomers [33]. Notably, a recent paper describing the crystal structure of PRRSV nsp1α, an arterivirus ortholog of the EAV ZF and PCPα domains, reported that the protein exists in equilibrium between monomers and dimers in solution. Residues from both subdomains also contribute both to nsp1α dimerization and the formation of a hydrophilic groove at the dimer surface [16].

### The quantitative balance among EAV mRNA species is critical for efficient virus production

**Figure 8:**
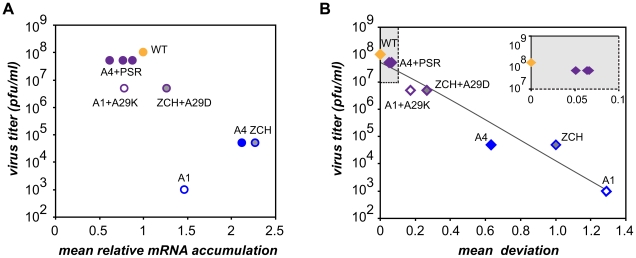
Relationship between viral mRNA accumulation profiles and infectious virus yield. The data obtained on the accumulation of genome and sg mRNAs were used for a quantitative assessment of nsp1 mutant phenotypes in terms of (A) changes in mRNA accumulation compared to wt and (B) the extent to which the balance between viral transcripts was disturbed (B). (A) For each mutant and pseudorevertant, a value for the accumulation of each of its 7 mRNAs at 11 h post-transfection had been assigned as compared to that of the wt control (see Fig. 4B, 7B and 7D). The mean of these seven values (“mean relative mRNA accumulation”) was plotted against the corresponding progeny virus titer in culture supernatants at 24 h post-transfection (Fig. 6B and table 2). Wild-type is depicted in orange. The engineered nsp1 mutants and nsp1 pseudorevertants are shown in blue and purple, respectively. The three pseudorevertants of mutant A4, displaying very similar phenotypes, are indicated as A4+PSR. (B) For each mRNA species, the deviation of its relative accumulation from the mean of the complete nested set of mRNAs (see panel A) was calculated. From these seven values, the mean (absolute) deviation was calculated for each mutant and plotted against virus titers as in (A). For wt, the mean deviation is 0. Engineered nsp1 mutants and their pseudorevertants are indicated as in (A). The data fit a negative exponential regression calculated using Microsoft Excel and depicted as a gray line (y = 6×10^6^+7e^−8.4306x^, R^2^ = 0.95). The inset shows an expanded view of the upper left quadrant of the graph (shaded in gray).

Characterization of the three nsp1 mutants with imbalanced viral mRNA accumulation profiles showed that the disruption of the balance was due to a reduction of the levels of certain viral mRNA species only for the A1 mutant (see Fig. 4B). By contrast, specific upregulation of most mRNAs was observed for both the ZCH and A4 mutants and the associated dramatic defects in virus production were surprising, also in view of the apparently undisturbed translation of viral mRNAs (Fig. 6A). In an attempt to quantify the relationship between virus yield and viral mRNA accumulation, we used the data sets of Fig. 4B, 7B, and 7D to calculate the mean relative mRNA accumulation for the ZCH, A1, and A4 mutants, as well as their pseudorevertants. Plotting these values versus the corresponding infectivity titers (derived from Fig. 6B and Table 3) revealed efficient production of infectious progeny for viruses with mRNA accumulation that was close to that of the wt control or modestly decreased (Fig. 8A). In contrast, severely reduced virus yields were observed when viral mRNA accumulation was increased, as seen for the ZCH, A1, and A4 mutants. This somewhat counterintuitive observation was rationalized by assessing the importance of the quantitative balance among EAV mRNA species for infectious virus production. In order to establish this relationship, for each mRNA species the absolute deviation of its relative accumulation from the mean of the complete nested set of mRNAs (Fig. 8A) was calculated. From these seven values, the mean (absolute) deviation was calculated for each mutant or pseudorevertant, and these values were also plotted against virus titers (Fig. 8B). By definition, the mean deviation is equal to zero for the wt virus, whose mRNA levels we refer to as “balanced”. All nsp1 mutants and their pseudorevertants have mean deviations larger than zero and these values reflect the magnitude of imbalance of their mRNA accumulation profiles. Remarkably, the plot revealed that the data nicely fit (R^2^ = 0.95) a negative exponential regression between infectious virus yield and mRNA imbalance, which is depicted as a negative linear regression in the semi-logarithmic plot of Fig. 8B. This strong relationship underlines that the magnitude of imbalance between different mRNA species, rather than the accumulation levels of mRNA species *per se*, is a chief factor affecting progeny yield. It should be noted that several factors may have affected our analysis of this relationship to a certain extent. For example, only a relatively small variety of nsp1 mutants and pseudorevertants was analyzed and although some pseudorevertants were recovered repeatedly, they were plotted only once. Also, the rapid emergence of pseudorevertants likely contributed to the virus titers measured for the ZCH, A1, and A4 mutants a 24 h post transfection, which would thus be overestimated in our analysis. Therefore, an extension of this study with new mutants and pseudorevertants may further refine the relationship illustrated by Fig. 8B. It would also be interesting to evaluate how virus yields are affected by a general imbalance between replication and transcription (A4 mutant) versus sg mRNA-specific changes (ZCH mutant), although the latter seem to have an added negative effect (A1 mutant).

The molecular basis of perturbed infectious particle production in the mutants is probably complex, although it is likely related to the altered relative abundances of viral structural proteins resulting from the respective changes in the levels of their mRNA templates (Fig. 4B and 6A). The observation that increased production of infectious progeny by the nsp1 pseudorevertants invariably correlated with restoration of balanced viral mRNA accumulation (Table 3 and Fig. 7) lends further support to this hypothesis. Differential changes in structural protein levels might adversely affect their ability to form complexes that drive particle assembly, or alter the stoichiometry of these complexes when incorporated in virions. The latter option is consistent with the observed decrease in relative specific infectivity of virion preparations from the ZCH, A1, and A4 mutants (Table 2). Detailed information on the architecture of EAV virions and the molecular interactions among EAV structural proteins that drive virus assembly is unfortunately lacking. Nevertheless, our data clearly establish a previously unknown link between balanced EAV mRNA accumulation and efficient virus production. We thus conclude that nsp1 critically promotes virion biogenesis by modulating viral mRNA accumulation, as well as acting at an additional, currently unknown step of the EAV replicative cycle, downstream of viral RNA synthesis (Fig. 3; [14])

### Nsp1 modulates a broad spectrum of successive stages in the nidovirus replicative cycle

The onset of +RNA gene expression is marked by translation of the viral genome. In subsequent stages of infection, this molecule is utilized also as the template for replication and, in some cases, transcription, as well as packaging into progeny virus particles. Virtually nothing is known about the temporal coordination of these distinct processes in the nidovirus replicative cycle. Non-structural protein expression is extensively regulated at the translational and post-translational level, by ribosomal frame-shifting and concerted autoproteolytic processing of replicase polyproteins, which together control the production of the core viral replicative enzymes [18],[19],[34],[35]. By contrast, regulation of structural protein expression is presumed to occur mainly at the transcriptional level, although the exact significance and control of the relative abundance of the various viral mRNAs in infected cells had not been examined for any nidovirus. Prior studies [13],[14], together with our present findings, clearly implicate EAV nsp1 in controlling the balance between replication and transcription (Fig. 3 and 4). Mutations in nsp1 were previously shown to selectively block or equally reduce the accumulation of all sg mRNA species. The pronounced upregulation of genomic RNA levels in nsp1 mutants with a complete block in sg mRNA production was also described before [14] and suggested to result from redirecting a limited pool of RNA-synthesizing complexes, normally engaged in both replication and transcription, to the exclusive amplification of the viral genome.

Some of the mutant phenotypes in this report, however, are poorly compatible with the above scenario. Genome RNA levels were increased 4–6 fold in the A1 and A4 mutants, for which sg mRNAs synthesis was clearly detectable and even enhanced (Fig. 4). In addition, the ZCH and A1 mutations differentially modulated the accumulation levels of a specific subset of sg mRNAs and their subgenome-length minus-strand templates (Fig. 4 and 5). This effect was accompanied by unchanged genome RNA levels in the ZCH mutant. Taken together, these data imply that nsp1 does not only allow the viral RdRp complex to engage in discontinuous minus-strand synthesis, but also enables it to differentiate between the various body TRS motifs it encounters while traversing the genomic template. This occurs by a currently unknown mechanism that is different from the previously described “polar attenuation” caused by the relative position of body TRSs in the array of successive attenuation signals that need to be “overcome” by the minus-strand RNA-synthesizing complex [36]. The changes in viral RNA accumulation observed for the A1 and A4 mutants could result from partial loss of recruitment of nsp1 to the RdRp complex, or its compromised ability to recognize RNA signals that direct discontinuous RNA synthesis. Viral RNA synthesis would then be shifted towards replication, but the increased availability of template and/or viral enzymes that this causes (Fig. 4B and 6A) might account for the relatively high levels of sg mRNA accumulation in these two mutants. This, in turn, would imply that the availability of nsp1 is important for its function in transcription. Indeed, the intracellular distribution of nsp1 is distinct from that of the other EAV nsps – a large fraction of the protein is present in the host cell nucleus [33], while only ∼25% of the cytoplasmic nsp1 fraction co-sediments with the membrane-bound viral RNA-synthesizing complexes upon their isolation from infected cells [37]. Immunofluorescence analysis did not reveal a significant change in intracellular nsp1 distribution for any of the mutants we described here (data not shown), but more rigorous biochemical studies are needed to ascertain the recruitment of nsp1 to RdRp complexes is completely unchanged. Nsp1 mutations clearly influenced minus-strand RNA accumulation (Fig. 5), although we cannot formally exclude that the protein controls minus-strand RNA stability rather than synthesis. Unfortunately, analysis of the kinetics of minus-strand accumulation in the ZCH and A1 mutants was hampered by the rapid emergence of pseudorevertants (Fig. 6B; Table 3) and the low abundance of these molecules at earlier time points post-transfection, which precluded their accurate quantitation (data not shown). Resolving this issue thus remains a formidable technical challenge. The recently described protocols for isolation of active viral RNA-synthesizing complexes from EAV-infected cells [37], as well as *in vitro* activity assays using a recombinant form of the EAV RdRp [38] might provide better platforms for future research on the mechanistic aspects of nsp1 function.

Nsp1 remains the only known arterivirus protein specifically implicated in the regulation of transcription [13],[39] and, to date, a functional counterpart has not been identified in coronaviruses or other nidoviruses. Species-specific changes in viral mRNA abundance were observed upon inactivation of the nsp14 exoribonuclease of human coronavirus 229E [40]. However, the major effect of this mutation was a reduction of the accumulation of all viral mRNAs by more than 100-fold. In view of the (indirect) dependence of transcription on genome replication and translation, the phenotype of the nsp14 mutants should be interpreted with caution. Nevertheless, a potential specific role of the coronavirus exonuclease in sg mRNA transcription deserves further investigation, although its analysis may be complicated by the multifunctionality of this protein, which was implicated in improving the fidelity of viral RNA synthesis [41],[42] and also includes an N7-methyltransferase domain [43]. Elegant studies of the tombusvirus replicase have shown that genome replication and sg mRNA synthesis can be effectively uncoupled by mutations in the C-terminus of the viral RdRp, which could only be achieved after separation of the protein-coding sequence from overlapping regulatory RNA sequences [44]. This example once again underlines the theoretical and technical challenges encountered while dissecting the complex mechanisms coordinating +RNA virus replication and transcription.

## Materials and Methods

### Cell lines

Baby hamster kidney cells (BHK-21; ATCC CCL10) were used for all experiments. The cells were maintained at 37°C in BHK-21 medium (Glasgow MEM; Invitrogen) supplemented with 5% fetal calf serum (FCS), 10% tryptose phosphate broth, 100 U/ml of penicillin, 100 µg/ml of streptomycin and 10 mM HEPES, pH = 7.4. Upon transfection or infection with wt or mutant EAV, BHK-21 cells were incubated at 39.5°C, since this elevated temperature shortens the replication time of the virus substantially without any adverse side effects [45].

### EAV reverse genetics

The substitutions in the nsp1-coding sequence listed in Table 1 and Table 3 were engineered using appropriate shuttle vectors and standard site-directed mutagenesis PCR [46]. Sequence analysis of the cloned fragments was used to verify the introduction of the appropriate nucleotide substitutions and exclude the presence of undesired mutations. The mutations in the nsp1-coding sequence were then transferred to pEAV211 or pEAN551, both derivatives of EAV full-length cDNA clone pEAV030 [45] containing some engineered restriction sites. Viruses derived from either pEAV211 or pEAN551 were previously shown to display a wt phenotype [17],[47]. The virus derived from the pEAV211 construct was used as a wt control in all experiments.

In vitro RNA transcription from *Xho*I-linearized wt or mutant EAV full-length cDNA clones was performed using the mMESSAGE mMACHINE T7 Kit (Ambion). Seven µg of in vitro-synthesized EAV RNA were electroporated into 3.5×10^6^ BHK-21 cells using the Amaxa Cell Line Nucleofector Kit T and the program T-020 of the Amaxa Nucleofector (Lonza) according to the manufacturer's instructions. Cells were resuspended in BHK-21 medium and subsequently seeded on coverslips for immunofluorescence analysis or in 6-well clusters for analysis of intracellular protein and RNA levels, as well as virus production.

### EAV infection and plaque assays

For EAV infection, subconfluent monolayers of BHK-21 cells were inoculated with transfected cell culture supernatant diluted in PBS-DEAE/2% FCS. Following incubation at 39.5°C for 1 h, the inoculum was removed, DMEM/2% FCS was added, and the cells were incubated at 39.5°C for 16–18 h. For virus titration, BHK-21 cells seeded in 6-well clusters were infected with serial 10-fold dilutions of supernatants harvested from transfected cells and then incubated under semi-solid overlays consisting of DMEM supplemented with 50 mM HEPES, pH = 7.4, 2% FCS and 1.2% Avicel (FMC BioPolymer) at 39.5°C for 72 h. The overlays were aspirated, cells were fixed with 8% formaldehyde in PBS, and stained with crystal violet. For plaque purification, a solid overlay of DMEM containing 50 mM HEPES, pH = 7.4, 2% FCS and 1% agarose was used.

### Immunofluorescence analysis

Immunofluorescence analysis of EAV-transfected cells was performed as described previously [48]. Briefly, cells were analyzed at 11 hour post-transfection by dual labeling with a rabbit antiserum recognizing EAV nsp3 [49] and an anti-N mouse monoclonal antibody (3E2; [50]). These proteins are expressed from RNA1 and RNA7, respectively. Nuclei were visualized for cell counting by staining with 1 µg/ml Hoechst 33258 (Sigma-Aldrich). Transfection efficiencies were determined at 11 h post-transfection by counting cells with the Scion Image software (Scion Corporation) and calculating the percentage of cells positive for EAV nsp3.

### RNA isolation and detection of EAV mRNAs by gel hybridization

Analysis of viral RNA accumulation was carried out before completion of the first replication cycle. Cells transfected with wt or mutant EAV derivatives were lysed at 11 h post-transfection, and total intracellular RNA was isolated by acid phenol extraction as previously described [25]. Viral mRNAs were detected by resolving total RNA in denaturing agarose-formaldehyde gels, and equal sample loading was confirmed by ethidium bromide staining of ribosomal RNA. The gels were subsequently dried and hybridized to a ^32^P-labeled probe (E154, 5′-TTGGTTCCTGGGTGGCTAATAACTACTT-3′) complementary to the 3′ end of the genome that recognizes both genome and all sg mRNAs [25]. The gels were exposed to phosphorimager screens, which were subsequently scanned using a Typhoon Variable Mode Imager (GE Healthcare). Image analysis and quantification of band intensities were performed with the ImageQuant TL software (GE Healthcare).

### Detection of EAV minus-strand RNAs by ribonuclease protection assays

A two-cycle ribonuclease (RNase) protection assay, adapted from [51] and [24], was used for the detection of EAV minus-strand RNAs. Total intracellular RNA isolated at 11 h post-transfection was dissolved in 10 µl of Hybridization Buffer III (RPA III Kit; Ambion), denatured for 3 min at 95°C and incubated for 16 h at 55°C. Samples were then digested with 5 U of RNase T1 (Ambion) per µg total RNA in 10 mM Tris pH = 7.5, 300 mM NaCl, 5 mM EDTA for 60 min at ambient temperature. Following proteinase K treatment and phenol∶chlorophorm extraction, 0.5 µg of yeast RNA per µg total RNA was added as a carrier. After ethanol precipitation, equal amounts of a radiolabelled probe (see below) were added to each sample and, following sample denaturation at 85°C for 5 min, hybridization was carried out at 55°C for 16 h. RNase digestion of unhybridized RNA was performed using the RPA III Kit according to the manufacturer's protocol. Protected fragments were resolved in 5% polyacrylamide/8M urea gels, which were dried and exposed to phosphorimager screens. Image analysis and quantification were performed as described above.

To generate probes for minus-strand detection, cDNA fragments derived from the EAV genome (nucleotides (nt) 3687–4013), RNA6 (nt 68–206 from the leader sequence and nt 11870–12057 from the body sequence) and RNA7 (nt 68–206 and nt 12252–12429) were inserted downstream of the T7 promoter in pcDNA3.1 using standard cloning procedures. Radiolabelled RNA transcripts were generated by in vitro transcription in the presence of [α-^32^P]CTP (Perkin Elmer) using MAXIscript T7 Kit (Ambion) and purified from 5% polyacrylamide/8M urea gels by elution for 3h at 37°C in 0.5 M NH_4_OAc, 0.2% SDS, 1mM EDTA. The transcript generated for detection of genomic minus strands –pRNA1, was 356 nt long and contained 327 nt of (−)RNA1-specific sequence. The transcript and EAV-specific sequence length were 382 nt and 327 nt, respectively, for the probe detecting (−)RNA6 (pRNA6), and 372 nt and 319 nt for the probe specific for (−)RNA7 (pRNA7). Detection of RNA1(−) was performed using sample RNA corresponding to approximately 1.25×10^4^ cells and 20 fmol of radiolabelled probe. Levels of (−)RNA6 and (−)RNA7 were determined in samples corresponding to 4×10^4^ and 2.5×10^4^ cells, respectively, using 5 fmol radiolabelled probe. These conditions ensured that the values obtained were in the linear range of the assays (data not shown).

### Protein analysis

Cells transfected with wt or mutant EAV derivatives were lysed at 11 h post-transfection as described previously [52]. The protein concentration in the lysates was determined using the Bio-Rad protein assay reagent. Equal amounts of total protein were subjected to SDS-PAGE and transferred to Hybond-P PVDF membrane (GE Healthcare) by semidry blotting. After blocking with 5% non-fat milk in PBS containing 0.5% Tween-20, the membranes were incubated with the following antibodies: anti-EAV nsp3 (see above), rabbit anti-M [52], anti-N (see above), or an anti-β-actin mouse monoclonal antibody (AC-74, Sigma), all diluted in PBS containing 5% non-fat milk, 0.5% bovine serum albumin and 0.5% Tween-20. HRP-conjugated secondary antibodies (DAKO) and an ECL-Plus kit (GE Healthcare) were used for detection.

### Reverse transcription and quantitative PCR

In order to determine relative specific infectivity, supernatants from BHK-21 cells transfected with wt or mutant EAV derivatives were harvested 24 h after transfection and clarified by low-speed centrifugation. Virions from 1 ml of clarified supernatant were purified by pelleting through a 0.4 ml cushion of 20% sucrose in 20 mM Tris pH = 7.5, 100 mM NaCl, 1 mM EDTA at 55,000 rpm for 45 min at 4°C using a TLS-55 rotor in a Beckman tabletop ultracentrifuge. Virion RNA was isolated from pellet fractions by acid phenol extraction as described above. Complementary DNAs were synthesized with Thermoscript Reverse Transcriptase (Invitrogen) using a primer complementary to a region in ORF1a of the EAV genome (EAV418as, 5′-AGCCGCACCTTCACATTG-3′). Quantitative PCR (qPCR) was performed essentially as previously described [53],[54]. Briefly, a cDNA aliquot was amplified with EAV-specific oligonucleotides EAV418as and EAV417s (5′ CATCTCTTGCTTTGCTCCTTAG-3′) using HotStar *Taq* Polymerase (Qiagen) and SYBR Green I (Molecular Probes) in an iCycler machine (Bio-Rad). The data obtained were analyzed with iCycler software, and the specificity of the reaction was confirmed by the melting curve of the amplified products. To generate a standard curve, serial ten-fold dilutions of the virion RNA sample derived from cells transfected with the wt EAV construct were reverse-transcribed and amplified by qPCR in parallel. The resulting standard curve had an R^2^ = 0.99 and a 6-log linear range for the EAV ORF1a amplicon (data not shown). The relative genomic RNA contents of virions produced by EAV mutants were calculated by comparing their threshold cycle (Ct) values against the standard curve and the resulting values were normalized to the wt genomic RNA content, which was set at 1. The relative specific infectivity of each EAV mutant was then determined by dividing the respective mutant∶wt pfu ratio by the mutant∶wt relative genomic RNA content.
